# Genetic Spectrum of Idiopathic Restrictive Cardiomyopathy Uncovered by Next-Generation Sequencing

**DOI:** 10.1371/journal.pone.0163362

**Published:** 2016-09-23

**Authors:** Anna Kostareva, Artem Kiselev, Alexandra Gudkova, Goar Frishman, Andreas Ruepp, Dmitrij Frishman, Natalia Smolina, Svetlana Tarnovskaya, Daniel Nilsson, Anna Zlotina, Tatiana Khodyuchenko, Tatiana Vershinina, Tatiana Pervunina, Alexandra Klyushina, Andrey Kozlenok, Gunnar Sjoberg, Irina Golovljova, Thomas Sejersen, Eugeniy Shlyakhto

**Affiliations:** 1 Almazov Federal Medical Research Centre, St. Petersburg, 197341, Russia; 2 Department of Women’s and Children’s Health and Centre for Molecular Medicine, Karolinska Institute, Stockholm, 17176, Sweden; 3 St. Petersburg State Polytechnical University, St. Petersburg, 195251, Russia; 4 Department of BioinformRatics, Wissenschaftszentrum Weihenstephan, Technische Universität München, 85354, Freising, Germany; 5 Institute of Bioinformatics and Systems Biology, Helmholtz Zentrum München, German Research Center for Environmental Health, 85764, Neuherberg, Germany; 6 Institutite of Molecular Medicine and Surgery, Karolinska Institute, Stockholm, 17176, Sweden; 7 Department of Faculty Therapy, Pavlov Medical University, St. Petersburg, 197022, Russia; 8 ITMO University, Institute of Translational Medicine, St. Petersburg, 197101, Russia; 9 National Institute for Health Development, Tallinn, Estonia; Indiana University, UNITED STATES

## Abstract

**Background:**

Cardiomyopathies represent a rare group of disorders often of genetic origin. While approximately 50% of genetic causes are known for other types of cardiomyopathies, the genetic spectrum of restrictive cardiomyopathy (RCM) is largely unknown. The aim of the present study was to identify the genetic background of idiopathic RCM and to compile the obtained genetic variants to the novel signalling pathways using *in silico* protein network analysis.

**Patients and Methods:**

We used Illumina MiSeq setup to screen for 108 cardiomyopathy and arrhythmia-associated genes in 24 patients with idiopathic RCM. Pathogenicity of genetic variants was classified according to American College of Medical Genetics and Genomics classification.

**Results:**

Pathogenic and likely-pathogenic variants were detected in 13 of 24 patients resulting in an overall genotype-positive rate of 54%. Half of the genotype-positive patients carried a combination of pathogenic, likely-pathogenic variants and variants of unknown significance. The most frequent combination included mutations in sarcomeric and cytoskeletal genes (38%). A bioinformatics approach underlined the mechanotransducing protein networks important for RCM pathogenesis.

**Conclusions:**

Multiple gene mutations were detected in half of the RCM cases, with a combination of sarcomeric and cytoskeletal gene mutations being the most common. Mutations of genes encoding sarcomeric, cytoskeletal, and Z-line-associated proteins appear to have a predominant role in the development of RCM.

## Introduction

Restrictive cardiomyopathy (RCM) is one of the rarest cardiac disorders with a very poor prognosis, and heart transplantation is the only long-term treatment option [1]. After exclusion of secondary causes such as AL-amyloidosis and irradiation, the aetiology of RCM is most often genetic. Due to the rare incidence rate of RCM, there has been limited description of its genetic causes when compared to other cardiomyopathies, especially hypertrophic cardiomyopathy (HCM), but also dilated cardiomyopathy (DCM). The list of RCM-associated genes includes sarcomeric and cytoskeletal genes often similar to those genes observed in HCM and DCM, but in total the genotyping success rate is quite low, corresponding approximately to 30% [2–9]. Importantly, the mechanisms underlying different cardiac phenotypes, resulting from mutations in the same genes, and the basis for intrafamilial variability of cardiomyopathy phenotypes, are poorly understood [10–13]. Considerable progress in the understanding of genetic causes of cardiomyopathies has recently become possible, because of the development of high throughput, massively parallel genetic sequencing methods, namely next-generation sequencing (NGS). Compared to the conventional Sanger sequencing, NGS allows the coverage of a much wider panel of genes including giant genes such as titin, and enables the expansion of analysis to genes associated with cardiac arrhythmias, neuromuscular disorders, and cardiomyopathy phenocopies [14,15]. Recently, this approach was successfully applied to unravel the spectrum of genetic causes in patients with HCM and DCM [16–18]. In spite of several reports on the application of NGS technology for the dissection of genetic causes of RCM in individual patients, to our knowledge, a systematic analysis of the genetic background in RCM has not yet been performed [5]. Thus, in our study, we aimed to analyse 24 cases of idiopathic RCM using an NGS approach, with a panel of 108 cardiomyopathy and arrhythmia-associated genes.

## Materials and Methods

### Patient cohort and clinical examination

The study included 24 individuals with RCM, hospitalised or treated in clinics of the Federal Almazov Medical Research Centre, St. Petersburg, or Astrid Lindgren’s Children Hospital, Karolinska University Hospital, Stockholm, during the period from 2003 to 2014. The study was performed according to the Declaration of Helsinki, and approval was obtained from Karolinska Institute Ethical Review Board and Almazov Medical Research Centre Ethical Committee. Written informed consent was obtained from all subjects and their representatives prior to investigation. On behalf of the children enrolled in the study written informed consent was obtained from the next of kin. The diagnosis was based on the WHO/International Society and Federation of Cardiology Task Force clinical criteria and classified according to the European Society of Cardiology classification of cardiomyopathies [19]. RCM was defined as the condition of a heart with restrictive ventricular physiology in the presence of normal or reduced diastolic volumes (of one or both ventricles), normal or reduced systolic volumes, and normal ventricular wall thickness. In paediatric patients (0–18 years), the diagnosis was based on echocardiography features of RCM such as atrial dilatation in combination with normal or nearly normal left ventricular size and preserved or nearly preserved systolic function (left ventricular end diastolic dimension z-score ≤ 3, left ventricular wall thickness z-score ≤ 3, and fractional shortening ≥ 0.25). In some patients, the increased left ventricular end diastolic pressure was further confirmed by cardiac catheterization. Patients with the neuromuscular phenotype were also included in the study. Acquired causes of RCM such as AL-amyloidosis, carcinoid, prior irradiation, and antitumor treatment were excluded, based on anamnesis, serum protein electrophoresis, bone marrow investigation, cardiac MRI, and heart biopsy, where necessary. Patients with transthyretin amyloidosis or constrictive pericarditis were not included in this study cohort. In cases of transformation of clinical phenotype and myocardial morphology over time, patients were included in the study, if defined criteria of RCM were present at disease onset or during the prolonged period, predominantly contributing to the clinical manifestation. All patients were examined by a dedicated team of clinical physiologists or paediatric cardiologists and underwent echocardiography with Doppler, 12-lead, Holter monitoring, and blood testing for creatine kinase (CK). In cases of elevated CK levels or other signs of muscle system involvement, a detailed neurological examination and electromyography were performed. To compare the spectrum of genetic variants, ten patients with early onset ventricular arrhythmias without diastolic dysfunction, ischemic heart disease, or structural cardiac abnormalities were genotyped using the same panel of genes.

### Design of the target gene panel

First, a list of cardiomyopathy- and channelopathy-associated genes was compiled from the literature, including 108 genes that are implicated in cardiomyopathies or inherited arrhythmia syndromes (S1 Table). To ensure comprehensive coverage of the target genes, we extracted all annotated coding regions based on genes and track data from RefSeq, Ensembl, CCDS, Gencode, VEGA, SNP, and CytoBand. The resulting target region covered 426,332 bp and was used as the input data for SureSelect (Agilent Technologies, Santa Clara, California, USA), to design the custom capture oligonucleotides for in-solution target enrichment. Manual optimisation was carried out to re-adjust capture oligonucleotides in regions with lower capture efficiency. In total, 19,956 capture probes mapping to 424,430 bp were synthesised (BED file with target region is available upon request).

### Gene enrichment and next-generation sequencing

DNA was purified from whole blood using a FlexiGene Kit following manufacturer’s recommendations, with an additional step of RNAase treatment (Qiagen, USA). The amount of genomic DNA in the samples was determined using a Qubit 2.0 fluorometer (Life Technologies; Carlsbad, CA) and a QuantiFluor fluorometer. The quality of the genomic DNA samples was determined using a Nanodrop 1000 (ThermoFisher Scientific; Wilmington, DE) and agarose electrophoresis with SybrGold (Life Technologies; Carlsbad, CA). Then, 200 ng of genomic DNA per patient was digested by 8 pairs of restriction enzymes (25 ng of DNA per restriction pair) provided by Haloplex custom target enrichment kit (Agilent; Waldbronn, Germany). Restriction quality was determined by measuring enrichment control DNA by a Bioanalyzer High Sensitivity DNA assay (Agilent; Waldbronn, Germany). The digested DNA was hybridised to the custom-designed Haloplex probes using a Veriti Thermal Cycler (Life Technologies; Carlsbad, CA) for 16 h. Herculase II Fusion DNA Polymerase (Agilent; Waldbronn, Germany) was used for amplification, when preparing the sequencing-library with the Haloplex Target Enrichment System (Agilent; Waldbronn, Germany). The PCR reaction was performed using the same equipment as in the hybridization step. The enrichment process was performed with a haloplex probe during the 16-h period. All clean-up steps were performed with the Agencourt AMPure XP PCR purification bead system (Beckman Coulter; Pasadena, CA). The targeted DNA was captured on magnetic beads, washed twice with 80% ethanol, and was subsequently dried at room temperature for 10 min. Library concentrations were measured using the Bioanalyzer High Sensitivity DNA assay (Agilent; Waldbronn, Germany). Different libraries with compatible barcodes were pooled in equal amounts and clustered at a concentration of 8 pM. Sequencing of 2 × 250 cycles was performed on MiSeq instruments using MiSeq Reagent Kit v2 chemistry (Illumina; San Diego, CA).

For all samples, alignment was performed using a Burrows-Wheeler Aligner and was called with the SNPPET tool (http://www.eposters.net/pdfs/snppet-a-fast-and-sensitive-algorithm-for-variant-detection-and-confirmation-from-targeted.pdf) as a part of the SureCall software (Agilent Technologies) [20]. As an alternative, output BAM files obtained from BWA were processed using the GATK (V.3.3–0; http://www.broadinstitute.org/) pipeline to increase reliability. BAM files were sorted and indexed with Picard tools (V.1.128; http://broadinstitute.github.io). Restriction enzyme fingerprints were clipped with GATK ClipReads (this step is required for correct allele balance); then BAM files were realigned and recalibrated against dbSNP 138 (NCBI) with GATK tools [21]. For the detailed pipeline see S1 File. In addition to the described pipeline, targeted NGS (OS-Seq) was performed for patients 5, 9, 15–18, 21, and 22, with Blueprint Genetics Heart Panel (http://blueprintgenetics.com).

Coverage metrics files were produced with SAMtools depth and were proceeded by a custom R script [22]. All samples were annotated using Annovar [23]. All disease-related genetic variants were successfully validated by Sanger sequencing as the gold standard sequencing technology. The positive prediction value (PPV) was calculated as the total number of true positive variants divided by the sum of true positives and true negatives based on Sanger sequencing verification.

### Variant classification

To estimate the pathogenic role of the identified genetic variants, we classified them according to ACMG guidelines [24]. All variants were checked according to population databases (1000G, ESP, and ExAC). Additionally, rare SNPs within frequency range 1:1000–1:10000 (MAF% 0.1–0.01) according to 1000G, GO-ESP, or ExAC 0.2 databases were analysed and demonstrated for each patient.

### Bioinformatics approach to predict damaging effect of missense mutations

Pathogenicity of missense variants was assessed based on the MetaSVM predictions obtained from the dbNSFP database [25]. MetaSVM is a support vector machine, which classifies amino acid substitutions as tolerated or damaging by incorporating deleteriousness scores produced by 10 individual algorithms—SIFT, PolyPhen-2 HDIV, PolyPhen-2 HVAR, GERP++, Mutation Taster, Mutation Assessor, FATHMM, LRT, SiPhy, and PhyloP.

### Protein network analysis

A disease interaction network was generated by manual curation using the CIDeR database [26]. Text-mining tools such as iHop, Chilibot, and EvidenceFinder were used for literature mining of Pubmed abstracts and PMC full text articles [27–29]. Proteins with RCM variants were analysed for their physical and regulatory interactions. All interactions from the protein network were manually curated and supported by experimental evidence from the scientific literature. If available, we used data obtained for cell types related to cardiac cells.

### Statistics

The difference in the distribution of genetic variants between the groups was statistically analyzed by means of the Fisher’s exact test to obtain a *P* value. A *P* value of less than 0.05 was considered significant. Odds ratios (OR) and 95% confidence intervals (95% CI) were calculated to express the strength of the association.

## Results

### Patient characteristics

The main clinical characteristics of the patient cohort are summarized in Table 1. The study cohort represents a mix of pediatric and adult patients with RCM, since the enrollment was without age limit. The mean age of disease presentation in our group was 23 years. Importantly, more than half of the patients developed first symptoms of the disease before age 18, including two infant cases. The median survival time from diagnosis until death or heart transplantation was 3 years, being longer in the pediatric group than in the adult (4 and 2 years, correspondingly). In 30% of patients a familial history of cardiomyopathy was reported. Of note, in some patients we observed a transformation with time of restrictive phenotype either to dilated cardiomyopathy, or to hypertrophic. Similar phenotypic variability was also observed in some familial cases of cardiomyopathy. Out of 8 patients with family history, 4 belonged to families in which cardiac disorders were represented also by other types of cardiomyopathy than that of the proband. Further, in four patients there were signs of neuromuscular disease in addition to RCM (patients 1, 18, 19, 22).

**Table 1:** Clinical characteristics of the study group.

Patient	Gender	Age of presentation	Family history	Phenotypic transformation or concomitant phenotypes	NM symptoms	Arrhythmias/ICD implanted	LA diameter (mm)	LVPW(mm)	EF%	PA pressure(mm Hg)	Outcome
1	M	16	+		CK elevation, neuropathy confirmed by EMG	AF, LQTS	57	11	n/a	38	Death at age 20
2	F	45	+			AF	61	10	65	40	Death at age 58
3	F	40	-			AF	63	10	n/a	47	Death at age 42
4	M	43	+				59	9	59	52	Death at age 45
5	M	15	-			WPW, short PQ	52 (z-score 5.2)		58	50	SCD at age 16
6	F	30	+	HCM in mother			58	10	69	28	alive, 49 y.o.
7	F	12	+	Transformation to DCM in 9 years after clinical manifestation of RCM, LVNC in mother			55 (z-score 6.09)	9	54	40	Hx age 23
8	F	31	-			AF, AV block, Sinus bradycardia	56	9	n/a	45	Hx age 38
9	F	31	+			AV block, SA block	59	11	71	47	Alive, Hx list
10	F	22	-			AF	58	9	n/a	38	Alive, 24 y.o.
11	M	45	-				61	11	74	46	Alive, 52 y.o.
12	F	54	-			AV block, AF	64	12	n/a	42	Death at age 56
13	F	52	-			AF	61	12	n/a	54	Death at age 53
14	M	8	-	Transformation to HCM with maintained restrictivity			23 (z-score 3.2)	4 (z-score 0.55)	72	32	Alive, 12 y.o.
15	F	6 month	-	Transformation to HCM with restrictive physiology			28 (z-score 3.8)	3.7 (z-score -0.2)	74	38	Alive, 5 y.o., Hx list
16	F	3	-			RBBB	35 (z-score 4.89)	5.3 (z-score 1.3)	65	45	Alive, 5 y.o.
17	M	28	+	HCM with restrictive physiology in son		AV block, SA	62	12	67	50	Death at age 54
18	M	11	+		Diffuse myopathic process confirmed by EMG	AF, SSS, sinus bradycardia, VT, LQTS, ICD implanted	43 (z-score 4.45)	6.8 (z-score 0.8)	61	27	Alive, 15 y.o.
19	M	15	-	Initial HCM phenotype with no clinical symptoms, transformation to RCM and HF	CK elevation, distal myopathy	AV block, SA block, VT, ICD implanted	56	9	59	43	Death at age 33
20	F	12	-				74	11	78	58	Death at age 23
21	F	8 month	-			AF, SA block	34 (z-score 5.5)	4.0 (z-score 0.47)	n/a	40	Death at age 1.5
22	M	8	-		Arthrogryposis	Arthrogryposis, scoliosis, myopathy	42 (z-score 3.81)	9	62	41	Alive, 18 y.o.
23	M	16	-				59	10	65	44	Alive, 20 y.o.
24	M	38	-				68	11	60	54	Alive, 46 y.o.

SA—sinoatrial, Hx–heart transplantation, AV–atrio-ventricular, AF–atrial fibrillation, CK–creatine kinase, EMG—electromyoghraphy, SSS–sick sinus syndrome, VT–ventricular tachycardia, LQTS–long QT syndrome, HCM–hypertrophic cardiomyopathy, RBBB–right bundle branch block, DCM–dilated cardiomyopathy, RCM–restrictive cardiomyopathy, LVNC–left ventricular non-compaction, M–male, F–female, LA–left atrium, ICD–implanted cardioverter-defibrillator, LVPW–left ventricular posterior wall, EF–ejection fraction, PA–pulmonary artery, y.o.–years old, WPW–Wolff-Parkinson-White-syndrome. n/a—not applicable because of AF.

### Sequencing quality and coverage data

The median value of the per-sample average read depth in the 426332 bp target region across the samples was 601. Combining all samples and taking the median value across all samples, 99.1% of the target region was covered to a depth of 15 or more, and 95.1% to a depth of 50 or more, 90.1% to a depth of 100 or more. The mean coverage over all 108 genes was as high as 667-fold. The PPV based on Sanger sequencing was 97.58% (95% CI = 94.88–100).

### Spectrum of genetic variants in patients with RCM

**Fig 1:**
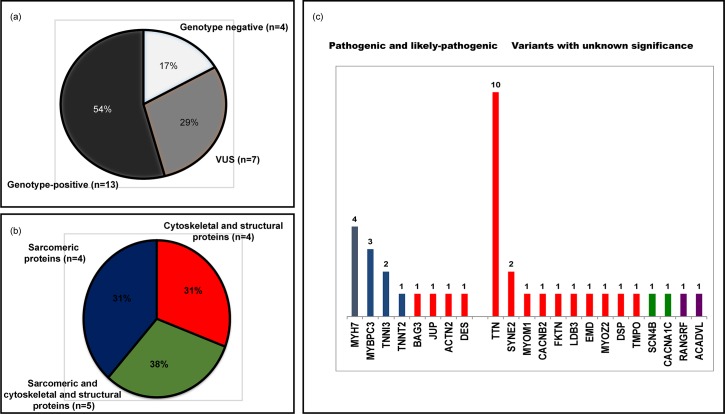
Genetic variants, identified in patients with RCM. (a) Overall yield of genotype-positive (pathogenic and likely pathogenic) variants and variants of unknown significance according to ACMG classification. (b) Genes where pathogenic, likely pathogenic variants and variants of unknown significance were detected. Blue corresponds to the genes encoding for sarcomeric proteins, red—to the genes encoding for cytoskeletal proteins, green—to ion channels and purple to the other genes. (c) Combination of pathogenic variants, likely pathogenic variants and variants of unknown significance in patients with RCMP.

In total we identified 5850 variants across the study group, on average 243±40 variants per patient. After annotation of all variants using ANNOVAR, we filtered out all common SNPs that resulted in 14 pathogenic or likely-pathogenic variants and 24 variants of unknown significance (Table 2, see S2 Table). Pathogenic and likely-pathogenic variants were identified in 8 out of 108 genes studied resulting in 54% of genotype-positive cases (13 patients, Fig 1A). Of 14 pathogenic and likely-pathogenic variants 10 were identified in genes encoding sarcomeric proteins (*MYH7*–4, *MYBPC3*–3, *TNNI3*–2 and *TNNT2*–1) and 4 in genes encoding structural and cytoskeletal proteins (*BAG3*, *JUP*, *ACTN2*, *DES*) (Fig 1B). Among 24 variants of unknown significance 19 were identified in structural and cytoskeletal genes (*TTN*, *SYNE*, *MYOM1*, *CACNB2*, *FKTN*, *LDB3*, *EMD*, *MYOZ*, *DSP*, *TMPO*), 2 in ion channel genes and 2 in genes encoding mitochondrial proteins. As expected, most of VUS were identified in *TTN* gene. Among 13 of genotype-positive patients in 31% the pathogenic or likely-pathogenic variant was detected within cytoskeletal genes (patients 1, 4, 8 and 19), and in 31% within sarcomeric genes (patients 2, 3, 17 and 20). In the remaining 5 patients (38%) pathogenic or likely-pathogenic variants in sarcomeric genes were associated by variants of unknown significance within cytoskeletal genes (Fig 1B).

**Table 2:** List of pathogenic, likely pathogenic and variants of unknown significance in patients with RCM.

	Pathogenic and likely-pathogenic variants						Variants of unknown significance				
Patient	Category	Gene	Position GRCh37Amino acid change	rs	MAF%	predicted effect	Gene	rs	Amino acid change	MAF%	predicted effect
1	P	BAG3	chr10:121431885:C>T NM_004281:**P209L**	rs121918312 [7]		D	TTN		chr2:179428256:T>C NM_001267550:**T27535A**	0.07	T
							MYOM1	rs376646252	chr18:3085111:C>T NM_003803:**G1424E**	0.002	D
2	LP	TNNT2	chr1:201332505:CTC>- NM_001001432:158_158del*			-					
3	P	MYH7	chr14:23894612:C>T NM_000257:**G768R**	rs727503260 [32]		D					
4	LP	JUP	chr17:39925402:G>A NM_021991:**R176W**	rs368336007 [10]	0.007	D	CACNB2		chr10:18787342:A>G NM_201597:**D131G**		D
5											
6	P	MYBPC3	chr11:47354816:TCCAC>- NM_000256: E1085fs*			-	FKTN		chr9:108377558:G>A NM_006731:781-1G>A*		-
7							TTN	rs375533809	chr2:179629287:C>TNM_133379:**V3319I**	0.003	T
8	LP	ACTN2	chr1:236889307:A>T NM_001278343:**N175Y**			D					
9							RANGRF	rs201464864	chr17:8192147:T>C NM_016492:**F14S**	0.007	D
							ACADVL		chr17:7121058:A>G NM_001270447:**I18V**		T
10											
11											
12	LP	MYH7	chr14:23893311:G>C NM_000257:**I909M**	rs377722048 [9]		D	LDB3	rs530979771	chr10:88441527:G>A NM_001171610:**R219Q**	0.004	T
							EMD		chrX:153608649:G>T NM_000117:**E107D**		T
13	P	MYBPC3	chr11:47353740:G>A NM_000256:**Q1233X**	rs397516037	0.0008	-	TTN		chr2:179445166:C>A NM_001267550:**D22314Y**	0.002	T
							TTN	rs72647879	chr2:179640343:C>A NM_133379:**R2083I**	0.0008	T
							TTN		chr2:179399559:T>C NM_001267550:**Q33928R**	0.0008	T
14	LP	MYBPC3	chr11:47371630:G>A NM_000256:**P147L**			T	MYOZ2		chr4:120072047:G>C NM_016599:**G33R**		D
	P	MYH7	Chr14:23894139:C>A NM_000257:**L840M**	rs730880747		D					
15							TTN		chr2:179441101:G>A NM_001267550:**T23253I**		D
16											
17	LP	MYH7	chr14:23894566:C>T NM_000257:R783H*	rs397516142 [8]		D					
18							SYNE2	rs548596262	chr14:64692150:C>T NM_182914:**S6877F**	0.01–0.0016	T
							DSP	rs730880090	chr6:7580808:G>T NM_004415:**S1462I**	0.006	T
							TTN		chr2:179482230:C>T NM_001267550:**S15861N**		T
19	P	DES	chr:220285069:G>A Splice site Exon 3+1G>A			-					
20	P	TNNI3	Chr19: 55665445:G>- NM_000363:del644fs			-					
21	P	TNNI3	chr:19:55665439:G>A ENSG00000129991:**R145W**			D	SCN4B		chr11:118015854:A>G NM_174934:**L51P**		D
							SYNE2		chr14:64496643:C>T NM_182914:**R2249X**	0.0016	-
22							TTN		chr:2:179414366:A>G NM_001267550:**S30695P**	0.0008	D
23							TTN		chr2:179474516:G>A NM_001267550:**L17212F**		D
24							TTN		chr2:179615440:G>A NM_133379:**A3896V**		T
							CACNA1C		chr12:2774110:T>C NM_199460:**F1499S**		D
							TMPO		chr12:98927898:AT>- NM_003276:**T621fs**		-

Variant classification was performed according to ACMG guidelines [24].

P- pathogenic, LP–likely pathogenic. VUS–variant of unknown significance. MAF%–Minor allele frequency according to 1000G, ESP, or ExAC. Predicted effect of the mutations according to MetaSVM: D–damaging, T–tolerated, N–neutral. For prediction algorithms see S2 Table.

*–variants confirmed by segregation data in family members. Predicted effect of the mutations according to MetaSVM: D–damaging, T–tolerated. For other prediction algorithms see S2 Table.

Additionally, for each patient we identified a list of rare SNPs with a population frequency of 1:1000–1:10 000, which resulted in another 39 variants (S3 Table). As expected, the highest number of variants was also found in the *TTN* gene (n = 16). No rare SNPs were detected in the group of sarcomeric protein genes or mitochondrial genes. In contrast, the number of variants in desmosomal and membrane-associated genes was significantly higher compared to the group of pathogenic, likely-pathogenic variant or variants of unknown significance (p = 0.003).

To investigate how the identified spectrum of RCM-associated genetic variants differs from the spectrum of the genetic variants identified in other cardiac disorders, we compared the spectrum of RCM-associated variants with variants identified in patients with early onset ventricular arrhythmias without diastolic dysfunction or structural cardiac abnormalities. Normal coronary angiography and young age at disease onset excluded an ischemic etiology of the arrhythmic disorders. In ten patients we identified 14 pathogenic or likely-pathogenic variants in ion-channel encoding genes, no pathogenic or likely-pathogenic variants were found in sarcomeric genes or cytoskeletal genes. Hence, the observed genetic spectrum of RCM-associated variants does not represent a random combination and differs from that of arrhythmic cardiac disorders.

### Major pathways involved in RCM pathogenesis

**Fig 2:**
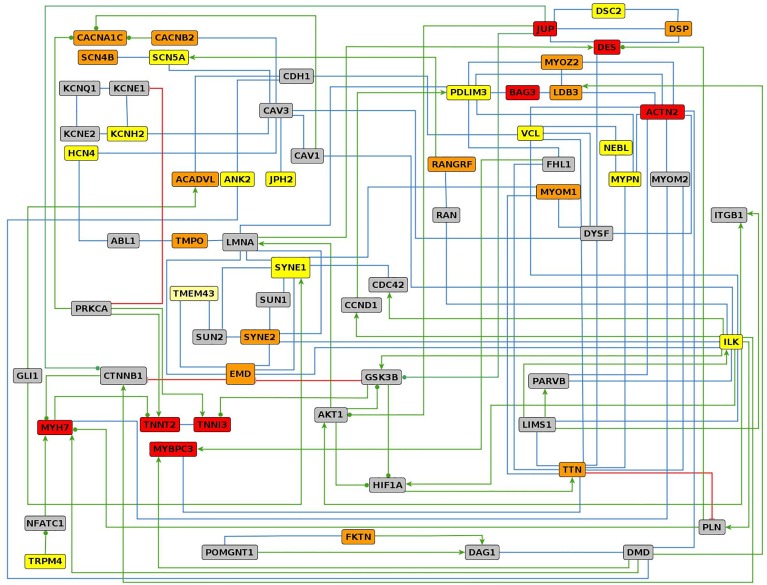
Interaction network of proteins harboring RCM-associated genetic variants. For proteins with RCM-associated pathogenic and likely pathogenic variants (red boxed), variants of unknown significance (orange boxes) and rare SNPs (yellow boxes) a closely interconnected network was generated by manual curation of scientific literature. The interlinking proteins are shown as gray boxes. Green arrows, red lines with cross bars, green lines with filled circles, and blue lines indicate activation, inhibition, modulation of activity, and direct physical interactions, respectively.

To understand the interaction landscape of the genes mutated in RCM patients, we created a tightly connected interaction network with 36 proteins corresponding to the genes described in this study (Fig 2). Including 30 interlinking proteins the network consists of 66 proteins connected by 124 interactions (S4 Table). We sought to use published data derived from cells and tissues that are related to cardiac cells. Out of the 124 physical and regulatory interactions considered here 38 and 33 were supported by experiments performed in cardiac and other types of muscle cells, respectively. Complementary interaction information was also found through protein structure analyses (6) and in vitro experiments (7). The largest group of interactions (79) is constituted by physical interactions between proteins, followed by regulatory interactions (34), protein modifications (10), and transport activities (1). Most proteins of the network belong to one of four functional groups: (i) sarcomeric proteins, (ii) mechanosensing and Z-line structures, (iii) nuclear membrane. The most interconnected protein is the plasma membrane protein ILK, which is involved in physical and regulatory interactions with 16 other proteins.

## Discussion

In the current study, we focused on the genetic spectrum of RCM using an NGS approach and demonstrated genotype-positive cases in 54% of patients. Our data in general correspond well to recently published study on another cohort of patients with RCM showing genotype-positive rate of 60% [30]. Previous publications on genetic causes of RCM have mainly addressed mutations in the sarcomeric genes, *MYH7*, *ACTC1*, *TNNI3*, and *TNNT2* as leading causes of RCM [31]. RCM has been characterised as a “severe form” of HCM, with a higher effect on Ca^++^ sensitivity and activity of actomyosin ATPase, because of specific functional properties of the mutations [32,33]. In our study, we have confirmed the role of sarcomeric proteins in the development of RCM, but have also extended the spectrum of pathogenic and likely-pathogenic variants underlining the role of cytoskeletal proteins in RCM pathogenesis.

The validation of pathogenic or likely-pathogenic variants is a very challenging task. Despite the increased number of variants recognized by high throughput sequencing technologies, many still remain of unknown significance, pending additional genetic or functional evidence. Many of such variants switch from being variants of unknown significance to likely-pathogenic or pathogenic variants, with the increasing number of reported patients and phenotypic descriptions. Most of previously reported pathogenic or likely-pathogenic variants were reported to reside within genes encoding sarcomeric proteins, which is in line with the data in our study. However, the majority of these previously reported variants in genes encoding sarcomeric proteins relate to the much more frequent HCM subgroup of cardiomyopathies. Considerably less is known about the genetic causes for RCM than for HCM, and interpretations of genetic results in RCM may be skewed if based on knowledge of genetic background in HCM. Therefore, even excluding genotype-positive cases, we consider all identified variants of unknown significance important for demonstration and analysis regardless of their predicted functional effect and presence in public databases.

Since approximately one half of the genotype-positive cases were associated with multiple pathogenic and likely-pathogenic variants, or variants of unknown significance, we speculate that, in some cases RCM might be the consequence of a combination of multiple variants, rather than resulting from one single disease-causing mutation. We propose that an individual combination of pathogenic and likely-pathogenic variants, variants of unknown significance, and rare SNPs underlies the intrafamilial phenotypic variability of RCM and the characteristic of being prone to phenotypic transformation.

Detected pathogenic and likely-pathogenic variants most frequently fall into the group of sarcomeric proteins, often in combination with variants of unknown significance in cytoskeletal and Z-line-associated proteins. Given the knowledge on participation of cytoskeletal and Z-line-associated proteins in force transmission, our data underscore the importance of mechanosensing and mechanotransducing proteins in the development of restrictive cardiac pathophysiology. We propose a model of RCM pathogenesis wherein sarcomeric or other gene mutations trigger a compensatory hypertrophic response such as in HCM, which cannot be fully implemented because of the concomitant defect in mechanotransducing proteins, resulting in a restrictive physiology. This hypothesis is further supported by bioinformatics modelling of major pathways involved in RCM, which has additionally highlighted the pivotal role of mechanosensing and mechanotransducing protein cascades.

Here we describe several new *MYBPC3* mutations associated with RCM. While *MYBPC3* mutations in HCM and DCM patients were described in numerous reports, only one mutation was previously reported as disease-causative in RCM [11,34–37]. This finding further underlines the genetic and pathophysiological link between HCM and RCM [38,39]. Additionally, we reveal several variants in genes, encoding ion channels and desmosomal proteins in patients with RCM. Whether these variants can affect myocardial relaxation is unknown. Recent studies using induced pluripotent stem cell-derived cardiomyocytes have identified alterations in cardiomyocyte contractility and prolonged relaxation time on a single-cell level caused by long QT syndrome-associated ion channel mutations [40]. The precise effect of these variants in the development of the RCM phenotype remains to be identified.

A major limitation of our study is the small number of patients included and the lack of segregation analysis, owing to the low number of family members and unavailability of these individuals for genetic testing. Such data might be of particular value in cases of multiple genetic variants in patients with RCM. Another limitation is the inability to infer a decisive conclusion regarding the functional effect of the multiple identified *TTN* variants. A high rate of spontaneous events owing to the huge size of *TTN* gene and difficulties of functional studies on giant muscle proteins makes it difficult to attribute a clear pathogenic effect to most of the identified variants. Additional information on the *TTN* variants identified from cardiomyopathy patients and healthy subjects will allow elucidation of the true roles of these variants in causing disease or their contribution to disease progression. Additionally, several genes recently reported in association with RCM such as *FLNC* were not included in the current analysis because the present study was undertaken prior to their identification as RCM associated genes [41]. The study of their role in RCM development will have a significant importance for further RCM research.

## Conclusion

In the present study we have identified a broad spectrum of RCM-associated variants using a next-generation sequencing approach and our findings underline the role of cytoskeletal genes in RCM development. Simultaneous screening of a wide panel of genes allowed us to identify multiple gene mutations in almost half of genotype-positive patients. We hypothesize that RCM is often triggered by a combination of multiple mutations, rather than by one disease-causing mutation. Our data further underscore the importance of mechanosensing and mechanotransducing proteins in the development of restrictive cardiac physiology.

## Supporting Information

S1 File Data analysis description. (DOCX)

S1 Table List of studied genes (Almazov_CardioMyoPathy_Arrhythmia_Noonan Design ID: 27291–1393420132). (DOCX)

S2 Table Prediction of mutation consequences by sequence-based computational methods. (DOCX)

S3 Table Rare SNPs identified in patients with RCM. (DOCX)

S4 Table List of the interactions with literature references. (XLSX)
