# Supplementary material for: Genetic Spectrum of Idiopathic Restrictive Cardiomyopathy Uncovered by Next-Generation Sequencing
Source: PLoS One. 2016 Sep 23;11(9):e0163362. doi: 10.1371/journal.pone.0163362 (PMC5035084; doi:10.1371/journal.pone.0163362)
Supplement: S1 File — (DOCX) [file pone.0163362.s001.docx]

**S1 File. Data analysis description.**

**GATK pipeline**

We used GATK V.3.3-0, and Picard tools ver. 1.119 & ver. 1.128 because some functions work in one version of the program but does not work in another.

In order to avoid unnecessary problems, use the references provided by GATK team (<ftp://ftp.broadinstitute.org/bundle/>). All steps were performed on a GNU Linux virtual machine running Debian 7.7 (QEMI or VirtualBox Image available upon request). For a detailed description of the used programs and parameters, see the GATK manual from <https://www.broadinstitute.org/>

1) **.bam file reordering** - This step is necessary if you do not use the same reference for alignment and calling.

/picard-tools-1.128/picard.jar \

ReorderSam \

INPUT= /workingfolder/BAMFILE.bam \

OUTPUT= /workingfolder/SortedBAMFILE.bam \

SORT_ORDER=coordinate \

2) **.bam file sorting** - Step required after reordering

/picard-tools-1.128/picard.jar \

SortSam \

INPUT= /workingfolder/BAMFILE.bam \

OUTPUT= /workingfolder/SortBAMFILE.bam \

SORT_ORDER=coordinate \

3) **.bam file indexing** - Step required after sorting

/picard-tools-1.119/BuildBamIndex.jar \

INPUT= /workingfolder/SortedBAMFILE.bam \

OUTPUT = /workingfolder/ReordSortBAMFILE.bam \

4) **Restriction enzymes fingerprint clipping** - step is necessary for the correct allelic balance

/GenomeAnalysisTK-3.3-0/GenomeAnalysisTK.jar \

--analysis_type ClipReads \

--outputStatistics /workingfolder/stat.txt \

--reference_sequence /hg19GATK/ucsc.hg19.fasta \

--input_file /workingfolder/ReordSortBAMFILE.bam \

--out /workingfolder/ClipReordSortBAMFILE.bam \

--cyclesToTrim "1-5" \

5) **Realign table creation**

/GenomeAnalysisTK-3.3-0/GenomeAnalysisTK.jar \

--analysis_type RealignerTargetCreator \

--reference_sequence /hg19GATK/ucsc.hg19.fasta \

--input_file /workingfolder/ClipReordSortBAMFILE.bam \

--out /workingfolder/INTERVALFILE.intervals \

--allow_potentially_misencoded_quality_scores \

6) **Local realignment**

/GenomeAnalysisTK-3.3-0/GenomeAnalysisTK.jar \

--analysis_type IndelRealigner \

--reference_sequence / hg19GATK/ucsc.hg19.fasta \

--input_file /workingfolder/ClipReordSortBAMFILE.bam \

-targetIntervals /workingfolder/INTERVALFILE.intervals \

--out /workingfolder/RlnClipReordSortBAMFILE.bam \

--allow_potentially_misencoded_quality_scores \

7) **Recalibration table creation**

/GenomeAnalysisTK-3.3-0/GenomeAnalysisTK.jar \

--analysis_type BaseRecalibrator \

--input_file /workingfolder/RlnClipReordSortBAMFILE.bam \

--reference_sequence /hg19GATK/ucsc.hg19.fasta \

-knownSites /hg19GATK/dbsnp_138.hg19.vcf \

--out /workingfolder/RECALFILE. table \

8) **Recalibrated .bam file printing**

/GenomeAnalysisTK-3.3-0/GenomeAnalysisTK.jar \

--analysis_type PrintReads \

--reference_sequence /hg19GATK/ucsc.hg19.fasta \

--input_file /workingfolder/RlnClipReordSortBAMFILE.bam \

-BQSR /workingfolder/RECALFILE. table \

--out /workingfolder/RclRlnClipReordSortBAMFILE.bam \

9) **SNV calling using GATK UnifiedGenotyper**

/GenomeAnalysisTK-3.3-0/GenomeAnalysisTK.jar \

--reference_sequence /hg19GATK/ucsc.hg19.fasta \

--analysis_type UnifiedGenotyper \

--dbsnp /data500gb/References/hg19GATK/dbsnp_138.hg19.vcf \

--input_file /workingfolder/RclRlnClipReordSortBAMFILE.bam \

--intervals /workingfolder/Haloplex_Regions.bed \ **#from Agilent SureSelect**

-filterMBQ \

--annotation AlleleBalance \

--out /workingfolder/VCF.vcf \

-stand_call_conf 30.0 \

-stand_emit_conf 10.0 \

--downsampling_type NONE \

--output_mode EMIT_VARIANTS_ONLY \

--genotype_likelihoods_model BOTH \

10) **Variants filtration**

GenomeAnalysisTK-3.3-0/GenomeAnalysisTK.jar \

--logging_level INFO \

--reference_sequence /hg19GATK/ucsc.hg19.fasta \

--analysis_type VariantFiltration \

--variant /workingfolder/VCF.vcf \

--out /workingfolder/**FilteredVCF.vcf** \

--clusterWindowSize 10 \

--clusterSize 3 \

--filterExpression "MQ0 >= 4 && (( MQ0 / (1.0 * DP )) > 0.1)" \

--filterName " HARD_TO_VALIDATE " \

--filterExpression "DP < 10" \

--filterName " LowCoverage " \

--filterExpression "QUAL < 30.0" \

--filterName " VeryLowQual " \

--filterExpression "QUAL > 30.0 && QUAL < 50.0" \

--filterName " LowQual " \

--filterExpression "QD < 2.0" \

--filterName " LowQD \

As a result you will have the **FilteredVCF.vcf** file which you can annotate using variable open source and commercial software.

11) **VCF annotation using Annovar**

**a) convert VCF to Annovar format**

/convert2annovar.pl \

-format vcf4 \

/workingfolder/**FilteredVCF.vcf \**

-outfile /workingfolder/**FilteredVCF.vcf**.avinput \

-allsample \

-withfreq \

**b) Variation annotation**

/table_annovar.pl \

/workingfolder/**FilteredVCF.vcf**.avinput \

/annovardbpath/humandb/ \

-buildver hg19 \

-out /workingfolder/**FilteredVCF.csv** \

-remove \

-protocol refGene,ensGene,knownGene,snp138,ljb26_all \

-operation g,g,g,f,f \

-nastring . \

-csvout \

After this step you will have Excel compatible CSV, which you can use for human friendly analysis.

**Prediction analysis**

1. Data collection and preprocessing

The dataset used in our analysis contains 77 amino acid substitutions in 35 proteins uncovered by next-generation sequencing in patients suffering from idiopathic restrictive cardiomyopathy. We identified protein IDs from the Entrez portal ^1^ using names of mRNAs annotated by the Annovar-2 software ^2^. Relevant FASTA-formatted protein sequences were retrieved from the NCBI Reference Sequence Database (RefSeq; ^3^).

2. Prediction of mutation effects

Amino acid substitutions were classified as damaging or neutral by the following sequence-based prediction methods:

1. SNPs&GO ^4^, implemented as a Support Vector Machine (SVM), classifies mutations into two categories – Neutral or Disease – based on a number of input features, including the information on mutation type, sequence neighborhood of the mutated residue, evolutionary conservation obtained from sequence profiles, and functional annotation defined by Gene Ontology ^4^. In addition SNP&GO also considers as input mutation deleteriousness predicted by the PANTHER algorithm ^5^.
2. PROVEAN (Protein Variation Effect Analyzer ^6^) predicts functional impact of single amino acid substitutions as well as insertions, deletions, and multiple substitutions based on sequence alignments.
3. Polyphen-2 ^7^ incorporates sequence conservation and predicts and protein structural properties (e.g. accessible surface area of amino acid residue) into a Naïve Bayes Framework.
4. SIFT ^8^ classifies mutations into Tolerated and Damaging by computing a combined score derived from the distribution of amino acid residues observed at a given position in the sequence alignment and the estimated unobserved frequencies of amino acids calculated from a Dirichlet mixture. To obtain sequence alignments target sequences were scanned against the UniRef90 database ^9^.
5. CADD (Combined Annotation–Dependent Depletion ^10^) is a support vector machine method for objectively integrating SIFT, PolyPhen-2, and GERP ^11^ annotations into a single measure (C score) for each variant. C-score correlates with allelic diversity, pathogenicity of both coding and non-coding variants, and experimentally measured regulatory effects. A C-score higher than 24 corresponds to a damaging mutation.
6. Mutation Assessor ^12^ captures the evolutionary conservation of a residue in a protein family and its subfamilies using combinatorial entropy measurement. It estimates a variant as functional (high, medium) or non-functional (low, neutral).

**References**

1. Database resources of the National Center for Biotechnology Information. *Nucleic Acids Res.* 2013;41(Database issue):D8-D20.

2. Wang K, Li M, Hakonarson H. ANNOVAR: functional annotation of genetic variants from high-throughput sequencing data. *Nucleic Acids Res.* 2010;38(16):e164.

3. Pruitt KD, Brown GR, Hiatt SM, et al. RefSeq: an update on mammalian reference sequences. *Nucleic Acids Res.* 2014;42(Database issue):D756-63.

4. Calabrese R, Capriotti E, Fariselli P, Martelli PL, Casadio R. Functional annotations improve the predictive score of human disease-related mutations in proteins. *Hum. Mutat.* 2009;30(8):1237-44.

5. Thomas PD, Campbell MJ, Kejariwal A, et al. PANTHER: a library of protein families and subfamilies indexed by function. *Genome Res.* 2003;13(9):2129-41.

6. Choi Y, Sims GE, Murphy S, Miller JR, Chan AP. Predicting the functional effect of amino acid substitutions and indels. *PLoS One* 2012;7(10):e46688.

7. Adzhubei I, Jordan DM, Sunyaev SR. Predicting functional effect of human missense mutations using PolyPhen-2. *Curr. Protoc. Hum. Genet.* 2013;(SUPPL.76):1-41.

8. Ng PC, Henikoff S. SIFT: Predicting amino acid changes that affect protein function. *Nucleic Acids Res.* 2003;31(13):3812-3814.

9. Suzek BE, Wang Y, Huang H, McGarvey PB, Wu CH. UniRef clusters: a comprehensive and scalable alternative for improving sequence similarity searches. *Bioinformatics* 2015;31(6):926-32.

10. Kircher M, Witten DM, Jain P, O’Roak BJ, Cooper GM, Shendure J. A general framework for estimating the relative pathogenicity of human genetic variants. *Nat. Genet.* 2014;46(3):310-5.

11. Cooper GM, Stone EA, Asimenos G, Green ED, Batzoglou S, Sidow A. Distribution and intensity of constraint in mammalian genomic sequence. *Genome Res.* 2005;15(7):901-13.

12. Reva B, Antipin Y, Sander C. Determinants of protein function revealed by combinatorial entropy optimization. *Genome Biol.* 2007;8(11):R232.
