# Supplementary material for: Genetic Spectrum of Idiopathic Restrictive Cardiomyopathy Uncovered by Next-Generation Sequencing
Source: PLoS One. 2016 Sep 23;11(9):e0163362. doi: 10.1371/journal.pone.0163362 (PMC5035084; doi:10.1371/journal.pone.0163362)
Supplement: S1 Table — (DOCX) [file pone.0163362.s002.docx]

**S1 Table. List of studied genes (Almazov_CardioMyoPathy_Arrhythmia_Noonan Design ID: 27291-1393420132).**

| **TargetID** | Interval | Cover. | High  Cover. | Low  Cover. |
| --- | --- | --- | --- | --- |
| **ABCC9** | chr12:21953968-22089618 | 100.00 | 41 | 0 |
| **ACTC1** | chr15:35082603-35087019 | 100.00 | 6 | 0 |
| **ACTN2** | chr1:236849964-236925929 | 99.82 | 23 | 0 |
| **AKAP9** | chr7:91570404-91739483 | 99.93 | 52 | 0 |
| **ANK2** | chr4:113825640-114309894 | 100.00 | 58 | 0 |
| **ANKRD1** | chr10:92672613-92680794 | 100.00 | 9 | 0 |
| **BAG3** | chr10:121411178-121436804 | 100.00 | 5 | 0 |
| **BRAF** | chr7:140426284-140624513 | 100.00 | 21 | 0 |
| **CACNA1C** | chr12:2162719-2800375 | 99.95 | 53 | 0 |
| **CACNA2D1** | chr7:81579698-82072785 | 100.00 | 42 | 0 |
| **CACNB2** | chr10:18429656-18828663 | 99.78 | 20 | 0 |
| **CALM1** | chr14:90863565-90871071 | 100.00 | 7 | 0 |
| **CALR3** | chr19:16589932-16606950 | 100.00 | 9 | 0 |
| **CASQ2** | chr1:116243852-116311172 | 100.00 | 11 | 0 |
| **CAV3** | chr3:8775553-8787563 | 100.00 | 2 | 0 |
| **CBL** | chr11:119077118-119170501 | 100.00 | 16 | 0 |
| **CRYAB** | chr11:111779478-111782458 | 100.00 | 3 | 0 |
| **CSRP3** | chr11:19204207-19214005 | 99.85 | 5 | 0 |
| **DES** | chr2:220283175-220290722 | 100.00 | 9 | 0 |
| **DMD** | chrX:31132798-33357392 | 99.92 | 88 | 0 |
| **DMPK** | chr19:46273729-46285639 | 100.00 | 16 | 0 |
| **DSC2** | chr18:28647971-28681944 | 100.00 | 18 | 0 |
| **DSG2** | chr18:29078205-29126716 | 100.00 | 15 | 0 |
| **DSP** | chr6:7542139-7586121 | 100.00 | 25 | 0 |
| **DTNA** | chr18:32335931-32470414 | 100.00 | 27 | 0 |
| **EMD** | chrX:153607835-153609567 | 100.00 | 6 | 0 |
| **EYA4** | chr6:133595909-133852374 | 100.00 | 21 | 0 |
| **FHL1** | chrX:135251952-135292194 | 100.00 | 10 | 0 |
| **FHL2** | chr2:105977730-106015563 | 100.00 | 8 | 0 |
| **FKTN** | chr9:108337304-108402414 | 99.68 | 12 | 0 |
| **FXN** | chr9:71650689-71714860 | 100.00 | 6 | 0 |
| **GAA** | chr17:78078376-78093140 | 100.00 | 19 | 0 |
| **GLA** | chrX:100652787-100662901 | 100.00 | 7 | 0 |
| **GPD1L** | chr3:32148194-32207412 | 100.00 | 8 | 0 |
| **HCN4** | chr15:73614812-73660621 | 100.00 | 8 | 0 |
| **HRAS** | chr11:532626-534332 | 100.00 | 5 | 0 |
| **ILK** | chr11:6625492-6631852 | 100.00 | 12 | 0 |
| **JPH2** | chr20:42743426-42815355 | 100.00 | 6 | 0 |
| **JUP** | chr17:39775836-39928116 | 100.00 | 20 | 0 |
| **KCND3** | chr1:112318689-112525358 | 100.00 | 7 | 0 |
| **KCNE1** | chr21:35821533-35821942 | 100.00 | 1 | 0 |
| **KCNE1L** | chrX:108867811-108868259 | 100.00 | 1 | 0 |
| **KCNE2** | chr21:35742768-35743159 | 100.00 | 1 | 0 |
| **KCNE3** | chr11:74168287-74168618 | 100.00 | 1 | 0 |
| **KCNH2** | chr7:150642443-150675011 | 100.00 | 16 | 0 |
| **KCNJ2** | chr17:68171171-68172474 | 100.00 | 1 | 0 |
| **KCNJ5** | chr11:128781159-128786636 | 100.00 | 2 | 0 |
| **KCNJ8** | chr12:21918647-21926560 | 100.00 | 2 | 0 |
| **KCNQ1** | chr11:2466319-2869243 | 100.00 | 18 | 0 |
| **KRAS** | chr12:25362719-25398328 | 100.00 | 5 | 0 |
| **LAMA4** | chr6:112430630-112575362 | 100.00 | 44 | 0 |
| **LAMP2** | chrX:119562329-119603034 | 100.00 | 11 | 0 |
| **LDB3** | chr10:88428439-88492743 | 100.00 | 16 | 0 |
| **LMNA** | chr1:156084700-156109640 | 99.71 | 16 | 1 |
| **MAP2K1** | chr15:66679676-66782963 | 100.00 | 11 | 0 |
| **MAP2K2** | chr19:4090586-4123882 | 100.00 | 11 | 0 |
| **MRPL3** | chr3:131181557-131221837 | 100.00 | 12 | 0 |
| **MYBPC3** | chr11:47353412-47374208 | 100.00 | 34 | 0 |
| **MYH6** | chr14:23851239-23876442 | 99.44 | 37 | 0 |
| **MYH7** | chr14:23882053-23902951 | 99.55 | 38 | 0 |
| **MYL2** | chr12:111348871-111358343 | 88.61 | 6 | 1 |
| **MYL3** | chr3:46899724-46904890 | 100.00 | 6 | 0 |
| **MYLK2** | chr20:30407374-30421610 | 100.00 | 12 | 0 |
| **MYOM1** | chr18:3067250-3215231 | 100.00 | 38 | 0 |
| **MYOZ2** | chr4:120057671-120107365 | 100.00 | 5 | 0 |
| **MYPN** | chr10:69866472-69970222 | 100.00 | 22 | 0 |
| **NEBL** | chr10:21074666-21462772 | 100.00 | 34 | 0 |
| **NEXN** | chr1:78381782-78408608 | 100.00 | 14 | 0 |
| **NF1** | chr17:29422318-29705959 | 99.63 | 59 | 1 |
| **NOS1AP** | chr1:162039958-162353331 | 100.00 | 13 | 0 |
| **NRAS** | chr1:115251146-115258791 | 100.00 | 4 | 0 |
| **PDLIM3** | chr4:186423438-186456598 | 99.45 | 9 | 0 |
| **PKP2** | chr12:32945348-33049675 | 100.00 | 14 | 0 |
| **PLN** | chr6:118880075-118880253 | 100.00 | 1 | 0 |
| **PRKAG2** | chr7:151254277-151573715 | 99.87 | 19 | 0 |
| **PSEN1** | chr14:73614718-73686007 | 100.00 | 11 | 0 |
| **PSEN2** | chr1:227068337-227083290 | 100.00 | 11 | 0 |
| **PTPN11** | chr12:112856906-112942578 | 100.00 | 16 | 0 |
| **RAF1** | chr3:12626003-12660230 | 99.57 | 17 | 0 |
| **RANGRF** | chr17:8192097-8193264 | 100.00 | 4 | 0 |
| **RBM20** | chr10:112404203-112595746 | 100.00 | 15 | 0 |
| **RYR2** | chr1:237205812-237995957 | 99.70 | 110 | 1 |
| **SCN1B** | chr19:35521715-35530615 | 99.64 | 5 | 0 |
| **SCN3B** | chr11:123504841-123524519 | 100.00 | 5 | 0 |
| **SCN4B** | chr11:118007732-118023398 | 100.00 | 5 | 0 |
| **SCN5A** | chr3:38591802-38674808 | 99.73 | 28 | 0 |
| **SCO2** | chr22:50962030-50962850 | 100.00 | 1 | 0 |
| **SDHA** | chr5:218461-256545 | 97.59 | 15 | 1 |
| **SGCD** | chr5:155756577-156186411 | 100.00 | 9 | 0 |
| **SHOC2** | chr10:112724107-112771586 | 100.00 | 8 | 0 |
| **SLC25A3** | chr12:98987747-98995316 | 100.00 | 8 | 0 |
| **SLMAP** | chr3:57743369-57913125 | 100.00 | 24 | 0 |
| **SNTA1** | chr20:31996303-32031436 | 100.00 | 8 | 0 |
| **SOS1** | chr2:39212955-39347573 | 99.65 | 23 | 1 |
| **SPRED1** | chr15:38545377-38643875 | 100.00 | 7 | 0 |
| **TAZ** | chrX:153640171-153649353 | 100.00 | 10 | 0 |
| **TCAP** | chr17:37821603-37822372 | 100.00 | 2 | 0 |
| **TGFB3** | chr14:76425520-76447246 | 100.00 | 7 | 0 |
| **TMEM43** | chr3:14166684-14183305 | 100.00 | 13 | 0 |
| **TMPO** | chr12:98909537-98941646 | 100.00 | 10 | 0 |
| **TNNC1** | chr3:52485281-52488041 | 100.00 | 6 | 0 |
| **TNNI3** | chr19:55663192-55668967 | 100.00 | 8 | 0 |
| **TNNT2** | chr1:201328328-201342392 | 100.00 | 21 | 0 |
| **TPM1** | chr15:63335019-63363381 | 100.00 | 16 | 0 |
| **TRDN** | chr6:123539736-123957930 | 98.85 | 48 | 1 |
| **TRPM4** | chr19:49661114-49714765 | 100.00 | 25 | 0 |
| **TTN** | chr2:179391729-179682294 | 98.80 | 346 | 18 |
| **VCL** | chr10:75757956-75877977 | 100.00 | 22 | 0 |
