# Supplementary material for: Genetic Spectrum of Idiopathic Restrictive Cardiomyopathy Uncovered by Next-Generation Sequencing
Source: PLoS One. 2016 Sep 23;11(9):e0163362. doi: 10.1371/journal.pone.0163362 (PMC5035084; doi:10.1371/journal.pone.0163362)
Supplement: S2 Table — (DOCX) [file pone.0163362.s003.docx]

**S2 Table. Prediction of mutation consequences by sequence-based computational methods.**

| **Gene** | **Mutation** | **SIFT^9^** | **Polyphen2HDIV^7^** | **Polyphen2HVAR^7^** | **LRT^1^** | **MutationTaster^11^** | **Mutation**  **Assessor^10^** | **FATHMM^12^** | **PROVEAN^2^** | **CADD^6^** | **GERP++^4^** | **PhyloP^3^** | **SiPhy^5^** | **dbNSFP^8^** |
| --- | --- | --- | --- | --- | --- | --- | --- | --- | --- | --- | --- | --- | --- | --- |
| ACADVL | I18V | T | T | T | . | T | . | D | T | 0.00 | -3.73 | -0.37 | 14.91 | **T** |
| ACTN2 | N175Y | D | D | D | D | D | H | T | D | 28.20 | 5.68 | 1.20 | 15.93 | **D** |
| ANK2 | Q2048R | D | P | P | T | T | M | T | T | 9.46 | 1.20 | 1.20 | 8.69 | **T** |
| ANK2 | S2064T | T | P | T | T | T | L | T | T | 0.18 | -1.99 | 0.96 | 6.51 | **T** |
| BAG3 | P209L | D | D | D | D | D | M | D | D | 34.00 | 6.17 | 0.93 | 20.88 | **D** |
| CACNA1C | F1499S | D | D | D | D | D | M | D | D | 31.00 | 4.07 | 0.96 | 13.52 | **D** |
| CACNA1C | A1736G | T | T | T | T | T | N | D | T | 21.70 | 3.72 | -0.67 | 10.79 | **T** |
| CACNA1C | T1953M | T | P | P | T | D | L | T | T | 22.40 | 4.26 | 0.85 | 16.92 | **T** |
| CACNB2 | D131G | D | D | D | D | D | M | D | D | 27.70 | 5.74 | 1.20 | 16.03 | **D** |
| DSP | S1462I | T | P | T | D | D | N | D | T | 22.80 | 5.65 | 1.05 | 13.95 | **T** |
| EMD | E107D | T | D | T | D | T | L | D | T | 13.46 | -0.27 | -0.28 | 7.78 | **T** |
| EYA4 | D327N | T | P | T | D | D | N | T | T | 22.50 | 5.67 | 1.05 | 19.76 | **T** |
| HCN4 | E1193Q | T | T | T | . | D | L | D | T | 22.70 | 3.52 | 0.81 | 13.19 | **D** |
| ILK | L53M | D | D | D | D | D | M | D | T | 24.00 | 3.88 | 0.05 | 11.45 | **D** |
| ILK | R211C | D | D | P | D | D | M | D | D | 34.00 | 5.40 | 0.93 | 18.34 | **D** |
| ILK | R211H | T | T | T | D | D | L | N | T | 22.80 | 4.45 | 1.05 | 15.44 | **D** |
| JPH2 | T286A | D | P | P | D | D | M | T | D | 24.20 | 2.98 | 0.71 | 11.26 | **T** |
| JUP | R176W | D | D | D | D | D | M | T | D | 25.20 | 4.82 | 1.04 | 15.31 | **D** |
| KCNH2 | L1045F | D | D | P | D | D | M | D | D | 23.90 | 4.97 | 0.97 | 15.75 | **D** |
| LDB3 | R219Q | T | D | D | T | D | L | T | T | 24.60 | 4.77 | 0.95 | 6.48 | **T** |
| MYBPC3 | Q1232X | . | . | . | . | D | . | . | . | 41.00 | 4.54 | 1.04 | 15.28 | **.** |
| MYBPC3 | P147L | D | T | T | . | T | N | T | T | 14.88 | 2.16 | 0.88 | 11.20 | **T** |
| MYH7 | I909M | D | D | D | . | D | M | D | T | 24.00 | 5.55 | 1.03 | 12.94 | **D** |
| MYH7 | R783H | T | P | P | . | T | L | D | T | 25.20 | 4.60 | 0.93 | 13.43 | **D** |
| MYH7 | G768R | D | D | D | . | D | M | D | D | 35.00 | 4.60 | 0.93 | 17.96 | **D** |
| MYH7 | L840M | D | D | D | . | D | H | D | T | 25.10 | 2.89 | 1.04 | 9.22 | **D** |
| MYOM1 | G1424E | D | D | D | D | D | M | T | D | 35.00 | 6.02 | 0.93 | 20.54 | **D** |
| MYOM1 | P396S | D | T | T | T | D | N | T | D | 24.40 | 5.55 | 1.05 | 19.69 | **T** |
| MYOZ2 | G33R | D | D | D | D | D | M | D | D | 28.90 | 5.75 | 0.14 | 18.72 | **D** |
| MYPN | D221V | T | D | P | T | D | N | T | T | 15.62 | 5.74 | 1.20 | 15.22 | **T** |
| NEBL | Y89X | . | . | . | D | D | . | . | . | 35.00 | 4.08 | -0.23 | 13.31 | **.** |
| PDLIM3 | S299N | T | T | T | T | D | N | D | T | 17.29 | 4.69 | 0.07 | 9.08 | **T** |
| RANGRF | F14S | D | D | D | D | D | M | T | D | 25.60 | 5.41 | 1.06 | 11.75 | **D** |
| SCN4B | L51P | D | D | D | D | D | M | D | D | 29.90 | 5.36 | 1.20 | 14.39 | **D** |
| SCN5A | P2006A | T | T | T | T | T | N | D | T | 0.00 | -2.61 | 0.95 | 4.48 | **T** |
| SYNE1B | L8619P | D | D | D | T | D | M | T | D | 32.00 | 5.61 | 1.19 | 15.80 | **T** |
| SYNE2 | R2249X | . | . | . | T | D | . | . | . | 37.00 | 3.34 | 0.93 | 9.53 | **.** |
| SYNE2 | S6877F | T | T | T | T | D | N | T | T | 23.40 | 5.99 | 0.85 | 10.75 | **T** |
| TMEM43 | R240C | D | D | D | D | D | M | T | D | 28.30 | 5.01 | 0.89 | 18.50 | **T** |
| TMPO | R274K | T | T | T | T | T | N | T | T | 0.31 | 2.57 | 0.95 | 8.30 | **T** |
| TNNI3 | R170W | D | D | D | D | D | M | D | D | 32.00 | -1.59 | 0.04 | 13.94 | **D** |
| TRPM4 | R250C | D | D | D | D | D | M | T | D | 29.40 | 4.49 | 0.86 | 16.54 | **D** |
| TRPM4 | W525X | . | . | . | . | D | . | . | . | 28.50 | -0.79 | 0.14 | 4.74 | **.** |
| TTN | R34840Q | D | D | D | . | D | N | T | D | 24.50 | 5.55 | 0.93 | 19.51 | **D** |
| TTN | Q33928R | D | T | T | . | D | L | T | T | 16.38 | 6.02 | 1.06 | 16.54 | **T** |
| TTN | A32765G | D | D | D | . | D | L | T | D | 21.70 | 6.17 | 1.05 | 20.88 | **T** |
| TTN | R32748C | D | D | D | . | D | M | T | D | 23.40 | 5.23 | 1.05 | 12.05 | **T** |
| TTN | S30695P | D | D | D | . | D | M | T | D | 22.00 | 5.74 | 1.20 | 16.34 | **D** |
| TTN | V29487M | D | P | T | . | D | N | T | T | 15.35 | 0.16 | 0.05 | 12.91 | **T** |
| TTN | T27535A | T | P | P | . | D | L | T | D | 13.49 | 4.80 | 1.07 | 12.75 | **T** |
| TTN | G26298R | T | D | P | . | D | L | T | D | 21.60 | 5.65 | 0.93 | 19.72 | **T** |
| TTN | S24535Y | D | D | D | . | D | M | T | D | 20.50 | 6.07 | 1.05 | 20.64 | **T** |
| TTN | T23253I | D | D | D | . | D | M | T | D | 15.22 | 5.74 | 1.05 | 19.93 | **D** |
| TTN | A22820P | D | D | D | . | D | H | T | D | 22.20 | 5.83 | 0.93 | 20.12 | **D** |
| TTN | D22314Y | D | D | D | . | D | M | T | D | 21.70 | 5.25 | 0.93 | 18.82 | **T** |
| TTN | E20241Q | D | D | D | . | D | M | T | T | 15.37 | 6.11 | 0.93 | 20.73 | **T** |
| TTN | L17212F | D | D | D | . | D | H | D | D | 19.64 | 5.85 | 0.95 | 13.37 | **D** |
| TTN | S15861N | D | D | D | . | D | M | T | T | 21.40 | 6.08 | 0.93 | 20.66 | **T** |
| TTN | P13156T | D | P | P | . | D | M | T | D | 15.93 | 5.22 | 0.95 | 10.72 | **T** |
| TTN | P12337S | . | . | . | . | T | . | T | . | 7.93 | 3.31 | 0.93 | 4.84 | **.** |
| TTN | H10092Y | T | T | T | . | T | L | T | D | 21.20 | 5.84 | 0.95 | 14.31 | **T** |
| TTN | D7462E | T | D | P | . | T | L | T | T | 10.46 | -1.51 | -0.53 | 12.15 | **T** |
| TTN | N6822S | T | T | T | . | T | L | T | T | 5.14 | 3.33 | 0.13 | 10.59 | **T** |
| TTN | Q5957H | D | P | P | . | T | L | T | D | 5.85 | 0.92 | 0.08 | 9.33 | **T** |
| TTN | D5516Y | D | D | D | . | D | M | D | D | 14.94 | 6.17 | 0.93 | 20.88 | **D** |
| TTN | A3896V | T | T | T | . | T | N | T | T | 18.12 | 1.77 | -0.09 | 5.96 | **T** |
| TTN | V3319I | T | T | T | . | T | L | T | T | 14.99 | 1.38 | 0.07 | 10.54 | **T** |
| TTN | R3120Q | T | D | P | . | D | L | T | T | 23.70 | 5.90 | 0.93 | 11.21 | **T** |
| TTN | R2083I | D | P | P | . | D | L | T | D | 14.85 | 3.54 | 0.01 | 11.58 | **T** |
| TTN | P12337S | . | . | . | . | T | . | T | . | 7.93 | 3.31 | 0.94 | 4.84 | **.** |
| VCL | H636R | T | D | D | D | D | M | T | D | 23.90 | 5.04 | 1.08 | 15.06 | **T** |

For SIFT, Polyphen2, LRT, Mutation Taster, Mutation Assessor, FATHMM, PROVEAN, and dbNSFP (MetaSVM, MetaLR) we used the following abbreviations: D – damaging; T – tolerated; P – possibly damaging; U – unknown; H – high; M – medium; L – low; N – neutral. The two ensemble scores from dbNSFP (MetaSVM and LR) were chosen as the main *in-silico* prediction methods based on ten component scores (SIFT, PolyPhen-2 HDIV, PolyPhen-2 HVAR, GERP++, MutationTaster, Mutation Assessor, FATHMM, LRT, SiPhy, PhyloP). Additionally, we present the CADD and PROVEAN results. The larger is the CADD score, the more likely it is that the SNP has a damaging effect. Larger GERP++, phyloP, and SiPhy scores correspond to more conserved sites. Missing data is designated as '.'.

**References**

1. Chun S, Fay JC. Identification of deleterious mutations within three human genomes. *Genome Res.* 2009;19(9):1553-1561.
2. Choi Y, Sims GE, Murphy S, Miller JR, Chan AP. Predicting the functional effect of amino acid substitutions and indels. *PLoS One* 2012;7(10):e46688.
3. Cooper GM, Stone EA, Asimenos G, Green ED, Batzoglou S, Sidow A. Distribution and intensity of constraint in mammalian genomic sequence. *Genome Res.* 2005;15(7):901-13.
4. Davydov E V, Goode DL, Sirota M, Cooper GM, Sidow A, Batzoglou S. Identifying a high fraction of the human genome to be under selective constraint using GERP++. *PLoS Comput. Biol.* 2010;6(12):e1001025.
5. Garber M, Guttman M, Clamp M, Zody MC, Friedman N, Xie X. Identifying novel constrained elements by exploiting biased substitution patterns. *Bioinformatics* 2009;25(12):i54-62.
6. Kircher M, Witten DM, Jain P, O’Roak BJ, Cooper GM, Shendure J. A general framework for estimating the relative pathogenicity of human genetic variants. *Nat. Genet.* 2014;46(3):310-5.
7. Kryukov G V, Pennacchio LA, Sunyaev SR. Most rare missense alleles are deleterious in humans: implications for complex disease and association studies. *Am. J. Hum. Genet.* 2007;80(4):727-39.
8. Liu X, Jian X, Boerwinkle E. dbNSFP v2.0: a database of human non-synonymous SNVs and their functional predictions and annotations. *Hum. Mutat.* 2013;34(9):E2393-402.
9. Ng PC, Henikoff S. SIFT: Predicting amino acid changes that affect protein function. *Nucleic Acids Res.* 2003;31(13):3812-3814.
10. Reva B, Antipin Y, Sander C. Determinants of protein function revealed by combinatorial entropy optimization. *Genome Biol.* 2007;8(11):R232. doi:10.1186/gb-2007-8-11-r232.
11. Schwarz JM, Rödelsperger C, Schuelke M, Seelow D. MutationTaster evaluates disease-causing potential of sequence alterations. *Nat. Methods* 2010;7(8):575-6.
12. Shihab HA, Gough J, Cooper DN, et al. Predicting the functional, molecular, and phenotypic consequences of amino acid substitutions using hidden Markov models. *Hum. Mutat.* 2013;34(1):57-65.
