# Supplementary material for: Genetic Spectrum of Idiopathic Restrictive Cardiomyopathy Uncovered by Next-Generation Sequencing
Source: PLoS One. 2016 Sep 23;11(9):e0163362. doi: 10.1371/journal.pone.0163362 (PMC5035084; doi:10.1371/journal.pone.0163362)
Supplement: S3 Table — (DOCX) [file pone.0163362.s004.docx]

**S3 Table. List of rare genetic variants (MAF 1:1000-1:10 000) in patients with RCM.**

|  | **SNP within frequency range 1:1000-1:10000 (MAF=0.1-0.01%)** | | | | |
| --- | --- | --- | --- | --- | --- |
| Patient | Gene | rs | Amino acid change | MAF% | predicted effect |
| 1 | TTN | rs200899806 | V29487M | 0.07 | T |
|  |  |  |  |  |  |
|  |  |  |  |  |  |
| 2 | TTN | rs181067357 | Q5957H | 0.023 | T |
| 3 | PDLIM3 | rs143812960 | S299N | 0.027-0.05 | T |
|  | ILK | rs200336608 | L53M | 0-0.03 | D |
|  | TTN | rs183482849 | D7462E | 0.008-0.05 | T |
|  | TTN | rs72648940 | D5516Y | 0.09 | D |
| 4 | VCL | rs71579374 | H636R | 0.05-0.09 | T |
|  | HCN4 | rs200507617 | E1193Q | 0.015 | D |
|  | TTN | rs72648273 | A32765G | 0.05-0.3 | T |
| 5 | KCNH2 | rs199473025 | L1045F | 0.02 | D |
|  | SCN5A | rs45489199 | P2006A | 0.09-0.1 | T |
| 6 | TTN | rs72648205 | G26298R | 0.05 -0.02 | T |
|  |  |  |  |  |  |
| 7 | CACNA1C | rs201777030 | T1953M | 0.03 | T |
| 8 | TRPM4 | rs71352737 | W525X | 0.07-0.09 | - |
|  | MYOM1 | rs554996810 | P396S | 0.014 | T |
| 9 | CACNA1C | rs369423529 | 1113+7A>G | 0-0.015 | - |
|  | TTN | rs200212521 | E20241Q | 0-0.09 | T |
| 10 | ANK2 | rs192761999 | S2064T | 0.02 | T |
|  | EYA4 | rs144415484 | D327N | 0.03 | T |
|  | TMEM43 | rs367910936 | R240C | 0.02 | T |
| 11 | ILK | rs200288502 | R211H | 0.01 | D |
|  | TTN | rs201474544 | P12337S | 0.02 | - |
| 12 | ILK | [rs140322345](http://www.ncbi.nlm.nih.gov/projects/SNP/snp_ref.cgi?rs=140322345) | R211C | 0.14-0.09 | D |
|  | JPH2 | rs144022614 | T286A | 0.04-0.1 | T |
|  |  |  |  |  |  |
| 13 |  |  |  |  |  |
|  |  |  |  |  |  |
|  |  |  |  |  |  |
|  |  |  |  |  |  |
| 14 |  |  |  |  |  |
|  |  |  |  |  |  |
|  |  |  |  |  |  |
| 15 | TRPM4 | rs144208673 | R250C | 0-0.05 | D |
|  | TTN | rs72646880 | A22820P | 0.09-0.22 | D |
| 16 | TTN | rs72648272 | R32748C | 0.09-0.29 | T |
|  | TTN | rs72650011 | H10092Y | 0.09-0.3 | T |
|  | TTN | rs72647894 | R3120Q | 0.09-0.3 | T |
| 17 |  |  |  |  |  |
| 18 | CACNA1C | rs201492706 | A1736G | 0.05-0.06 | T |
|  |  |  |  |  |  |
|  | TTN | rs201804005 | S24535Y | 0.01-0.03 | T |
| 19 | NEBL | rs147622517 | Y89X | 0.05-0.13 | - |
| 20 |  |  |  |  |  |
| 21 | ANK2 | rs201628725 | Q2048R | 0-0.02 0.008 | T |
|  | MYPN | rs185841477 | D221V | 0.07-0.01 | T |
|  | TTN | rs72650064 | P13156T | 0.03-0.07 | T |
| 22 | SYNE1B | rs139834542 | L8619P | 0.05-0.1 | D |
| 23 | TTN | rs199710082 | R34840Q | 0.016 | D |
|  | TTN | rs371518764 | N6822S | 0.016 | T |
| 24 | TMPO | rs139700737 | R274K | 0.07-0.09 | T |
|  |  |  |  |  |  |
|  |  |  |  |  |  |

MAF%–Minor allele frequency according to 1000G, ESP, or ExAC.

Predicted effect of the mutations according to MetaSVM: D–damaging, T – tolerated. For other prediction algorithms see S2 Table.
